# Association of Direct Oral Anticoagulants vs. Vitamin K Antagonists With Fractures in Atrial Fibrillation Patients: A Systematic Review and Meta-Analysis

**DOI:** 10.3389/fcvm.2021.713187

**Published:** 2021-07-22

**Authors:** Xiaojuan Wu, Linyan Hu, Jinjin Liu, Qiuping Gu

**Affiliations:** ^1^Department of Gastroenterology, Ganzhou People's Hospital, Ganzhou, China; ^2^Hengshui Health School, Hengshui, China; ^3^Department of Oncology, Ganzhou People's Hospital, Ganzhou, China

**Keywords:** atrial fibrillation, non-vitamin K antagonist oral anticoagulants, warfarin, fracture, meta

## Abstract

**Background:** Current evidence regarding the application of direct oral anticoagulants (DOACs) vs. vitamin K antagonists (VKAs) on the fracture risk is inconsistent. Therefore, we conducted a meta-analysis to evaluate the fracture risk of DOACs vs. VKAs in patients with atrial fibrillation (AF).

**Methods:** The PubMed and Embase databases were systematically searched until June 2021 for all the studies that reported oral anticoagulants in AF patients. The random-effect model with an inverse variance method was selected to pool the risk ratios (RRs) and 95% confidence intervals (CIs).

**Results:** A total of 10 studies were included in this meta-analysis. Among AF patients receiving anticoagulants, DOAC users showed a reduced risk of any fracture compared to those with VKAs (RR = 0.80; 95% CI: 0.70–0.91) regardless of gender [males (RR = 0.79; 95% CI: 0.67–0.92) and females (RR = 0.71; 95% CI: 0.57–0.89)]. Apixaban (RR = 0.75; 95% CI: 0.60–0.92) and rivaroxaban (RR = 0.73; 95% CI: 0.61–0.88), but not dabigatran and edoxaban, were associated with a decreased risk of any fracture compared with VKAs. DOAC users had decreased risks of osteoporotic fractures (RR = 0.63; 95% CI: 0.47–0.84) and hip/pelvic fractures (RR = 0.88; 95% CI: 0.79–0.97) compared to those treated with VKAs.

**Conclusions:** Our meta-analysis suggested that the use of DOACs was associated with a reduced risk of any fracture compared with VKAs. Further studies should confirm our findings.

## Introduction

Atrial fibrillation (AF) is becoming an aging-related disease, and osteoporotic fractures are major health threats in the elderly. Oral anticoagulants are widely used for thromboprophylaxis in AF patients for decades. Vitamin K antagonists (VKAs) such as warfarin has been speculated to increase the risk of osteoporotic fracture. Warfarin interrupts the vitamin K-dependent calcium balance and synergy with vitamin D bone-forming actions. Warfarin inhibits the γ-carboxylation of several osteoblast-specific proteins (1, 2), leading to low bone density and increased fracture risk (3). These observations propose a link between warfarin use and the risk of osteoporotic fractures (4, 5).

Direct oral anticoagulants (DOACs) including thrombin or Xa factor inhibitors (dabigatran, apixaban, rivaroxaban, and edoxaban) are recommended as the first-line drugs for thromboprophylaxis in AF patients. Data from both randomized controlled trials (RCTs) (4–7) and observational studies (8, 9) have shown that DOACs are at least non-inferior to warfarin for stroke prevention in AF patients. Additionally, DOACs might be associated with better outcomes in the elderly (10), as well as AF patients with complications [e.g., stroke (11), cancer, and peripheral artery disease (12)].

Since DOACs have no impact on osteocalcin, their effects on bone fracture have yet been undefined. A prior meta-analysis based on the RCTs (13) showed that DOACs were associated with a relatively lower fracture risk over warfarin in patients with AF or venous thromboembolism. However, there is a lack of consistent evidence regarding this issue in real-world settings. Several real-world studies found that there was no difference in the risk of bone fracture between DOACs and warfarin (14, 15), whereas other studies suggested that DOACs were associated with a lower risk of fracture compared to warfarin (16–18). Therefore, this meta-analysis was performed to compare the risk of fractures between DOACs vs. VKAs in AF patients.

## Methods

The meta-analysis was performed under the recommendations of the Cochrane handbook for systematic reviews (19) and the Preferred Reporting Items for Reporting Systematic Reviews and Meta-analyses (20). The data of the current study are available from the corresponding author on reasonable requests. We did not provide ethical approval because only the published data were included.

### Eligibility Criteria

In this study, the following inclusion criteria were applied: (1) population (P)-nonvalvular AF patients; (2) intervention (I) and control (C)-DOACs vs. VKAs; (3) outcome (O)-bone fractures including any fracture, major osteoporotic fractures, vertebral, and humerus/forearm/wrist fractures and hip/pelvic fractures; and (4) study design-RCTs or observational studies. The effect estimates were propensity score-matched or adjusted risk ratios (RRs) and 95% confidence intervals (CIs). Studies with no data, such as reviews, case reports, case series, editorials, guidelines, and conference abstracts, were excluded.

### Literature Search

The PubMed and Embase electronic databases were systematically searched from January 2009 (since the first available DOAC-dabigatran was applied to AF patients) to June 2021 for studies that compared the risk of any fracture between DOACs vs. VKAs in AF patients. The search strategy combined three kinds of search terms using the Boolean operator “and”: (1) *atrial fibrillation* OR *atrial flutter*, AND (2) *non-vitamin K antagonist oral anticoagulants* OR *NOACs* OR *direct oral anticoagulants* OR *DOACs* OR *dabigatran* OR *rivaroxaban* OR *apixaban* OR *edoxaban*, AND (3) *vitamin K antagonists OR warfarin OR coumadin OR phenprocoumon OR acenocoumarol*, AND (4) *fracture* OR *bone fracture* OR *osteoporosis* OR *osteoporosis fracture*. There were no linguistic restrictions in the literature search. The literature search strategy is shown in Supplementary Table 1. To ensure a comprehensive literature search, the reference lists of the retrieved studies were screened to identify the additional reports.

### Study Selection and Data Extraction

All the retrieved studies were screened by two reviewers independently. Potential eligible studies were chosen after reviewing the titles and abstracts based on the established inclusion and exclusion criteria. The disputable issues were resolved by consensus, or by a discussion with the third author.

The following information was collected including the first author and publication year, country, data source, study design, baseline data of the participants (sample size, age, and the sex), inclusion period, type of DOACs, the follow-up time of DOAC users, and type of fractures.

### Risk of Bias Assessment

For the *post-hoc* analysis of RCTs, the bias risks were evaluated according to the Cochrane risk of bias assessment tool (19). The bias risk of each study was scored as “low,” “unclear,” or “high” risk in each section. The “low risk” was defined when three out of five biases were “low” (21). The Newcastle-Ottawa Scale (NOS) tool was applied to evaluate the methodological quality of observational studies. A study with a NOS score of <6 was defined as low quality (22).

### Statistical Analysis

In this meta-analysis, we performed all the statistical analyses using the Stata software (version 15.0, Stata Corp LP, College Station, TX) and the Review Manager 5.3 software (the Nordic Cochrane Center, Rigshospitalet, Denmark). The Cochrane Q test and *I*^*2*^ statistic were the most commonly reported statistical methods to assess the heterogeneity, where *P* < 0.1 and *I*^*2*^ > 50% suggested substantial heterogeneity, respectively. The natural logarithms of RRs and standard errors of the included studies were calculated and then pooled by a random-effects model using an inverse variance method. The publication bias was assessed using the funnel plots, and further calculated using the Egger's and Begg's tests. The subgroup analyses were performed based on the DOAC types (dabigatran, apixaban, rivaroxaban, and edoxaban), the individual position of fractures (hip/pelvic fracture and osteoporosis fracture), gender (males vs. females), and length of the follow-up period (≥1 vs. <1 year).

## Results

### Study Selection

The process for electronic retrievals is shown in Supplementary Figure 1. A total of 10 studies [one sub-analysis of RCT (23) and nine observational studies (14–17, 24–28)] were included in this meta-analysis. To show the reliability of all the included studies, baseline information of the study participants is shown in Table 1. Six studies (15–17, 25–27) had a follow-up time of ≥1 year, 2 studies (14, 24) showed a follow-up time of <1 year, and two studies did not provide the specific follow-up time (23, 28).

**Table 1:** Baseline characteristics of the included studies of this study.

**Included studies**	**Country**	**Study** **design**	**Number of** **participants**	**Age** **(y)** **/Male (%)**	**Study** **period**	**Data** **source**	**DOACs**	**Follow-up in DOAC users (months)**	**Controls**	**Outcomes**	**Quality** assessment^*^
He-2020	Québec Canada	Observational study	25,663	75.6/50.3	2000–2014	Québec healthcare databases	DA; RIV; API	NA	VKAs	Any fracure,hip fracture, upper extremity fracture, vertebral fracture, Osteoporosis with pathological fracture	8
Lau-2020	Hong Kong China	Observational study	23,515	74.4/52.0	2010–2017	Clinical data analysis and reporting system	DA; RIV; API	14.1	warfarin	Osteoporotic fracture	8
Huang-2020	Taiwan China	Observational study	19,414	71.9/59.0	2012–2017	Taiwan's national health insurance research database	DA; RIV; API	28.8	warfarin	Hip, vertebral, and humerus/forearm/wrist fractures	8
Binding-2019	Danish	Observational study	37,350	NA/57.8	2013–2017	Danish national patient register	DOACs	24.0	warfarin	any fracture, major osteoporotic fractures, initiating osteoporotic medication, hip fractures	8
Lutsey-2019	United States	Observational study	167,275	68.9/62	2010–2015	MarketScan commercial claims and encounters and marketscan Medicare Supplemental and Coordination of Benefitsdatabases	DA; RIV; API	16.9	warfarin	Hip fractures, Inpatient fractures, All fractures	8
Chan YH-2019	Taiwan China	Observational study	24,338	74.6/56.8	2012–2017	National health insurance research database	EDO	>12.0	warfarin	Any fracture	8
Norby-2017	United States	Observational study	77,991	70.3/60.5	2010–2014	The truven health marketscan^®^ commercial claims and encounters database and the medicare supplemental and coordination of benefits database	RIV	12.0	warfarin	Hip/pelvic fracture	8
Lucenteforte-2017	Denmark	Observational study	16,850	NA/51.1	2009–2015	Danish national prescription registry	DA	12.6	warfarin	Any fracture	8
Bengtson-2017	United States	Observational study	61,648	70.1/63.3	2009–2012	The truven health marketscan^®^ commercial claims and encounters database and the medicare supplemental and coordination of benefits database	DA	15.0	warfarin	Hip/pelvic fracture	8
Steffel-2016	United States	*Post-hoc* analysis	20,205	72.0/62.4	NA	ENGAGE AF-TIMI 48 trial	EDO	NA	warfarin	Any fracture	Low risk

*ENGAGE AF-TIMI 48, effective anticoagulation with factor xa next generation in atrial fibrillation-thrombolysis in myocardial infarction 48; DOACs, direct oral anticoagulants; DA, dabigatran; RIV, rivaroxaban; API, apixaban; EDO, edoxaban; VKAs, vitamin K antagonists; NOS, newcastle-ottawa scale; NA, not available*.

* *The Newcastle-Ottawa Scale (NOS) items were used to evaluate the quality of the observational studies, which involve the selection of cohorts, the comparability of cohorts, and the assessment of the outcome)*.

We did the quality assessment and found that the sub-analysis of RCT (23) had a low risk of bias, and all of the included observational studies (14–17, 24–28) had an acceptable quality.

### DOACs vs. VKAs on the Risk of Fracture

**Figure 1:**
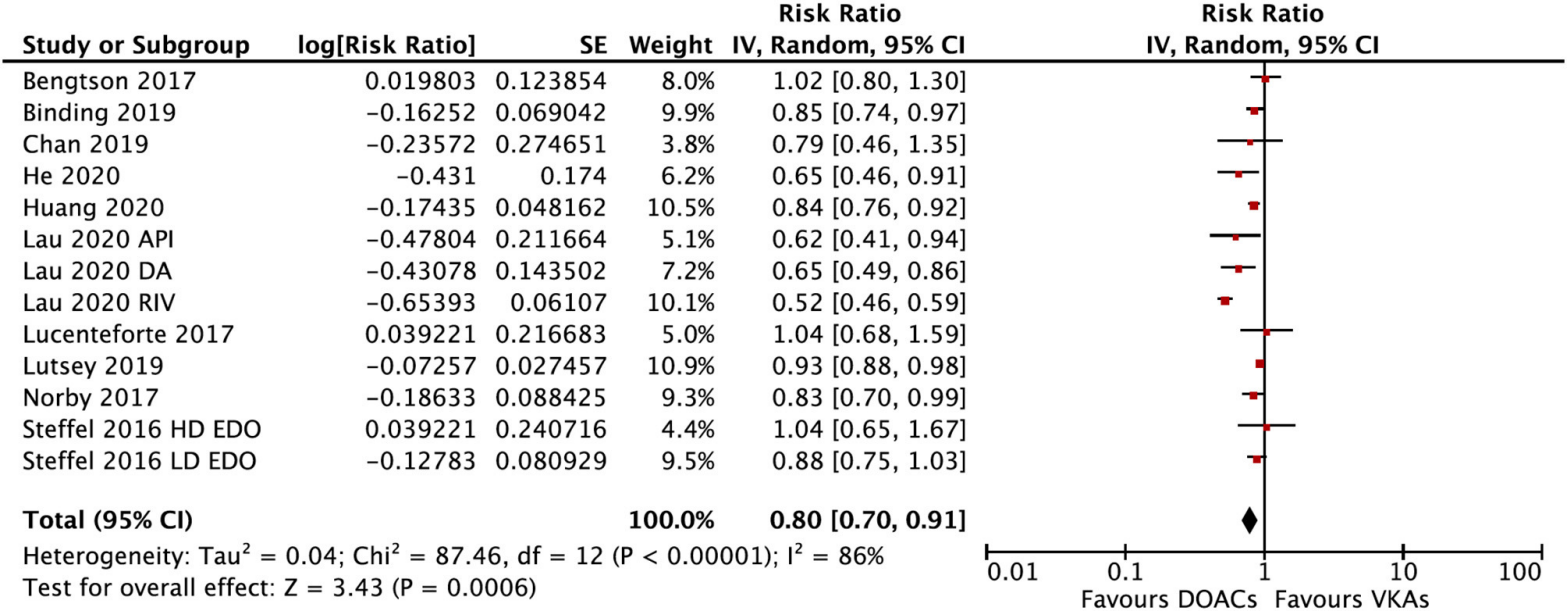
Comparing the risk of any fracture of DOACs vs. VKAs in AF patients. AF, atrial fibrillation; DOACs, direct oral anticoagulants; VKAs, vitamin K antagonists; DA, dabigatran; RIV, rivaroxaban; API, apixaban; EDO, edoxaban; CI, confidence interval; HD, high dose; LD, low dose; SE, standard error; IV, inverse of the variance.

The overall RRs and 95% CIs of fracture risks between DOACs vs. VKAs in AF patients are summarized in Supplementary Table 2. In the pooled analysis, compared with VKA use, the use of DOACs was associated with a decreased risk of any fracture (HR = 0.80, 95% CI 0.70–0.91) (Figure 1).

**Figure 2:**
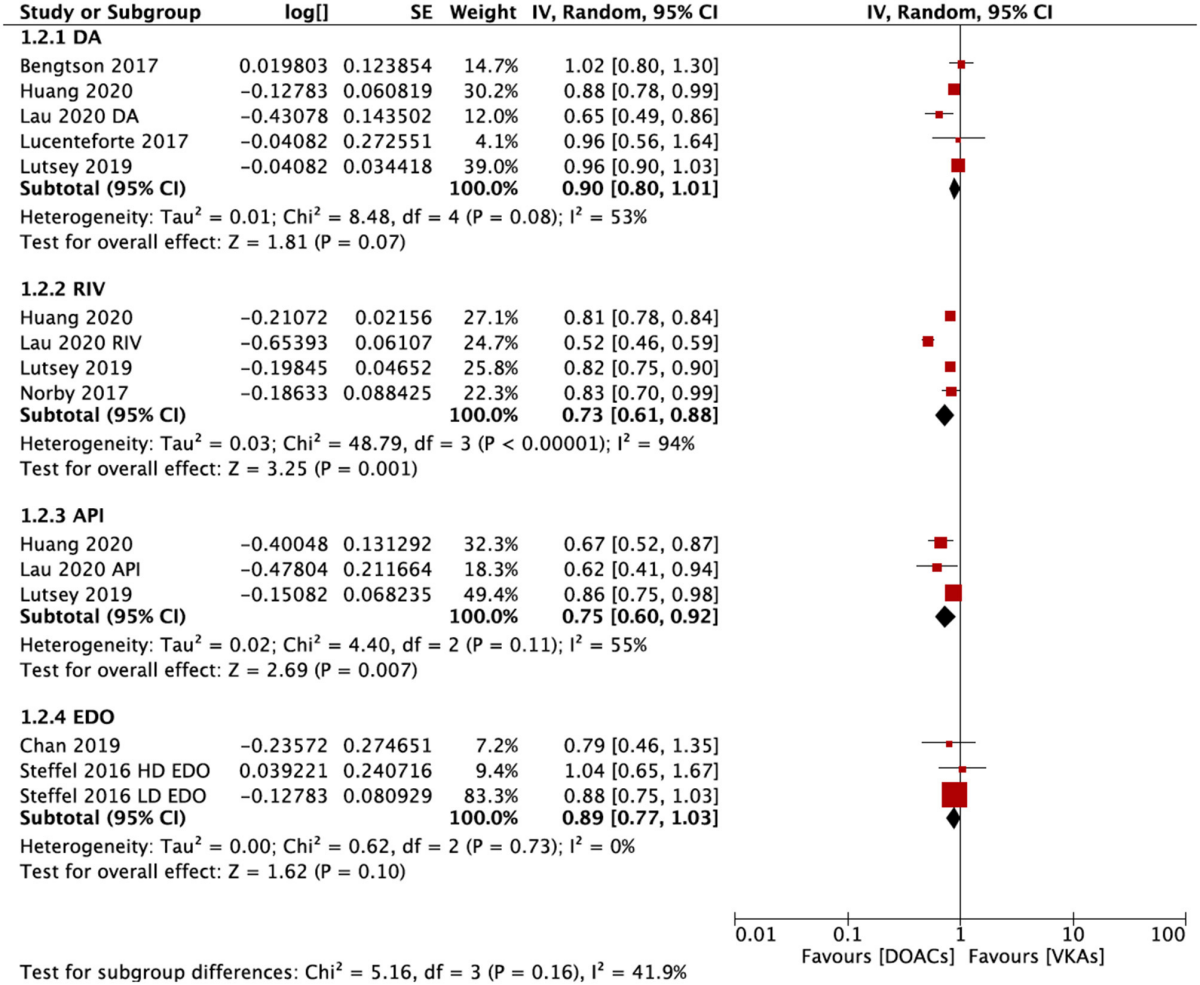
Subgroup analysis based on different types of DOACs regarding the risk of fractures of DOACs vs. VKAs in AF patients. AF, atrial fibrillation; DOACs, direct oral anticoagulants; VKAs, vitamin K antagonists; DA, dabigatran; RIV, rivaroxaban; API, apixaban; EDO, edoxaban; CI, confidence interval; SE, standard error; IV, inverse of the variance.

**Figure 3:**
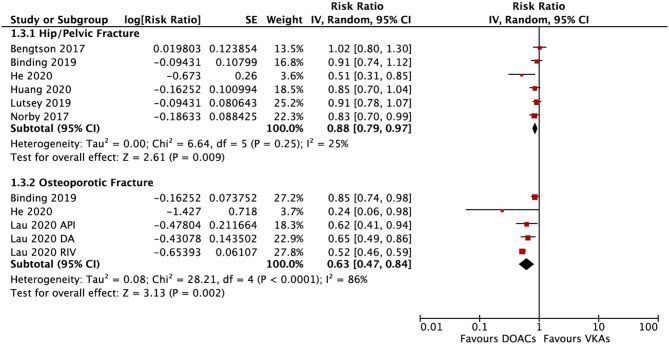
Subgroup analysis based on different positions regarding the risk of fractures of DOACs vs. VKAs in AF patients. AF, atrial fibrillation; DOACs, direct oral anticoagulants; VKAs, vitamin K antagonists; DA, dabigatran; RIV, rivaroxaban; API, apixaban; CI, confidence interval; SE, standard error; IV, inverse of the variance.

In the subgroup analysis based on the DOAC types, compared with VKAs, rivaroxaban (RR = 0.73; 95% CI: 0.61–0.88) and apixaban (RR = 0.75; 95% CI: 0.60–0.92), but not dabigatran (RR = 0.90; 95% CI: 0.80–1.01) and edoxaban (RR = 0.89; 95% CI: 0.77–1.03), were associated with a lower risk of any fracture (Figure 2). Compared with VKAs, the usage of DOACs acquired a lower risk of hip/pelvic fracture (RR = 0.88; 95% CI: 0.79–0.97) and osteoporosis fracture (RR = 0.63; 95% CI: 0.47–0.84) (Figure 3).

**Figure 4:**
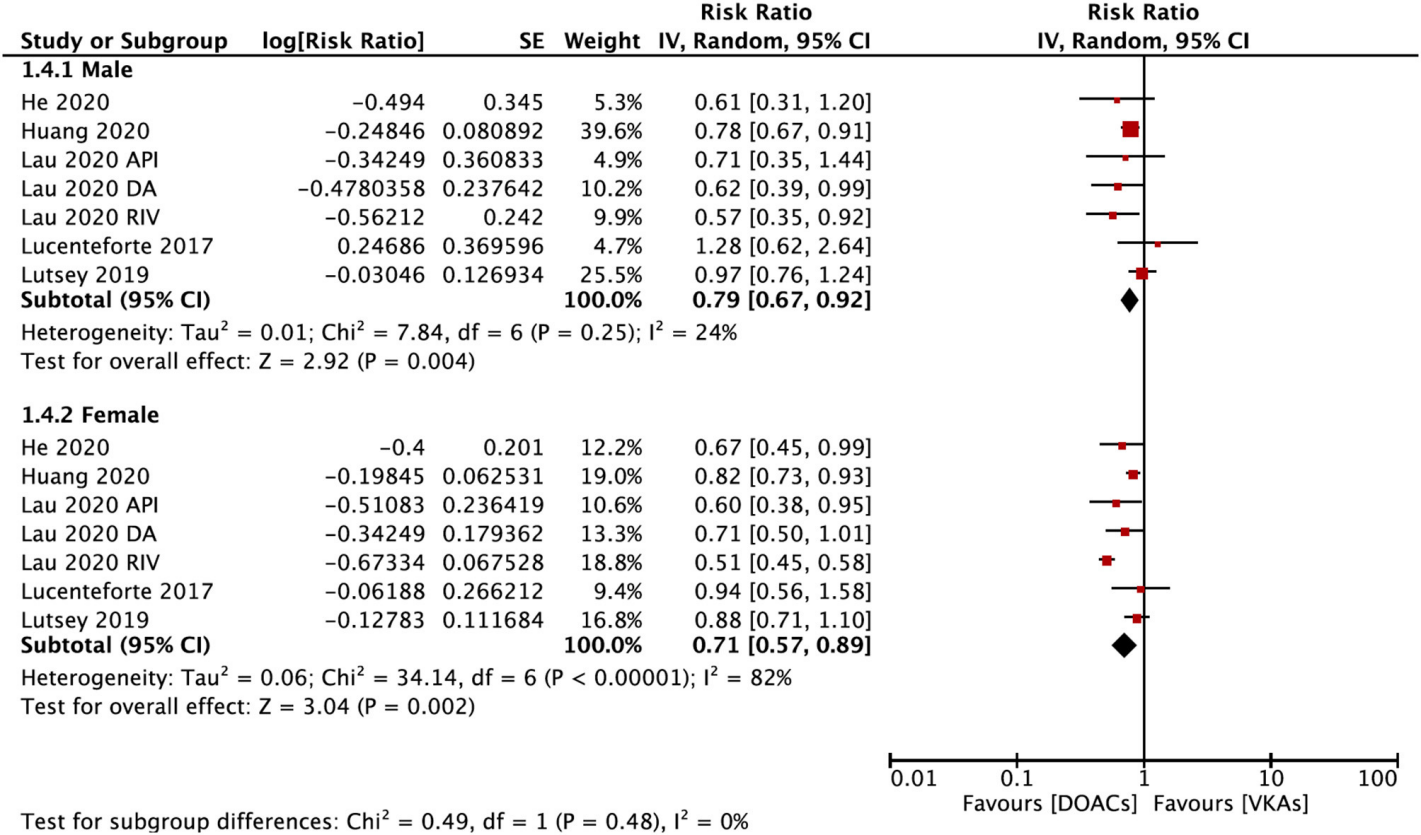
Subgroup analysis based on sex regarding the risk of fractures of DOACs vs. VKAs in AF patients. AF, atrial fibrillation; DOACs, direct oral anticoagulants; VKAs, vitamin K antagonists; DA, dabigatran; RIV, rivaroxaban; API, apixaban; CI, confidence interval; SE, standard error; IV, inverse of the variance.

**Figure 5:**
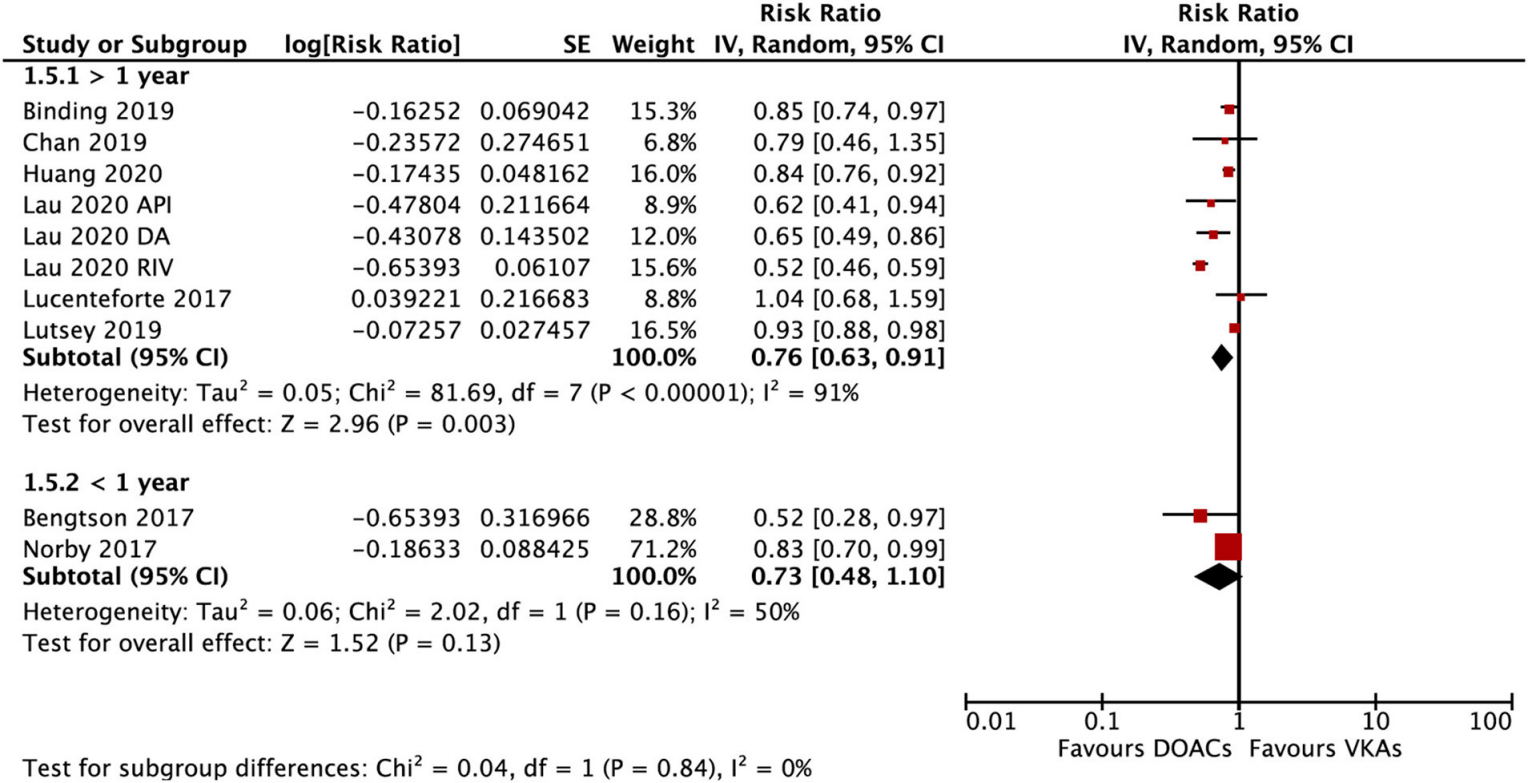
Subgroup analysis based on the follow-up time regarding the risk of fractures of DOACs vs. VKAs in AF patients. AF, atrial fibrillation; DOACs, direct oral anticoagulants; DA, dabigatran; RIV, rivaroxaban; API, apixaban; CI, confidence interval; SE, standard error; IV, inverse of the variance.

The subgroup analysis based on gender suggested that DOACs were associated with a lower risk of fractures in both males (RR = 0.79; 95% CI: 0.67–0.92) and females (RR: 0.71; 95% CI: 0.57–0.89) compared with VKAs (*P*_*interation*_ = 0.48; Figure 4). DOACs vs. VKAs were associated with a decreased risk of any fracture in patients with a follow-up of ≥1 year (RR = 0.76, 95% CI 0.63–0.91), but not in the group of <1 year (RR, 0.73, 95% CI 0.48–1.10), although the interaction was not significant between the two subgroups (*P*_*interaction*_ = 0.84; Figure 5).

### Publication Bias

No potential publication biases were found checked by the funnel plots (Supplementary Figure 2) combined with the Egger's (*P* = 0.479, Supplementary Figure 3) and Begg's (*p* = 0.837) tests.

## Discussion

In the present meta-analysis, compared with VKAs, the use of DOACs (mainly rivaroxaban and apixaban) was associated with a lower fracture risk among long-term AF patients. There was no significant different interaction between male and female patients. Overall, DOACs might be a safe alternative among AF patients in terms of decreasing the fracture risks compared with VKAs regardless of gender.

The potential increased risk of fracture with warfarin is coherent with the mechanism of anticoagulation. By regulating vitamin K, warfarin inhibits the γ-carboxylation of osteocalcin, which is associated with a low bone mineral density. Two prior meta-analyses assessed the risk of fracture associated with DOACs compared with VKAs (13, 29). One meta-analysis comprising 89,549 patients of 12 RCTs demonstrated that rivaroxaban and apixaban showed a lower fracture risk when compared to warfarin (13), consistent with our current findings. An *in vivo* study indicated that dabigatran has a better bone safety profile than warfarin because warfarin could interrupt bone by reducing the trabecular size and increasing bone turnover (30). Nevertheless, dabigatran has non-inferiority or superiority to warfarin in terms of reducing the fracture risk in the real-world population. Lutsey et al. found that the estimates between dabigatran and warfarin were near the null for hip and all clinical fractures (17). They only found some evidence of a lower risk of fractures requiring hospitalization associated with dabigatran (17). Lucenteforte et al. also presented no significant difference in the fracture risk between dabigatran with warfarin (25). In contrast, a retrospective cohort study published in 2020 reported a significantly lower risk of osteoporotic fractures associated with the use of dabigatran among AF patients (26). One potential explanation for the discrepancies across the studies may be the different definitions of fracture and the duration of oral anticoagulants. The biological effect of warfarin on bone metabolism is cumulative and chronic. Lucenteforte et al. restricted cohort eligibility to patients who had been continuously exposed to oral anticoagulants within 1 year, which might lead to an underestimation of fracture risk in warfarin users (25).

Edoxaban has no effects on the production of Gla-osteocalcin; and thus may have a lower risk of adverse effects on bone health in the rats (31). Although there are still no experiments on humans, evidence of the fracture risk with edoxaban use is limited. A *post-hoc* analysis from the ENGAGE AF-TIMI 48 trial showed that edoxaban has a comparable risk of fracture with warfarin irrespective of the dosage (23). Given the limited number of edoxaban-associated studies included in the meta-analysis, further study should confirm the fracture risk of edoxaban vs. warfarin in AF patients.

In the current meta-analysis, DOACs were showed a decreased risk of overall fracture events comparing with VKAs. Particularly, rivaroxaban and apixaban are showed reduced risks of fracture events. Although Lau et al. (26) did comparisons between dabigatran and rivaroxaban regarding the osteoporotic fractures risk in AF patients, no significant difference was detected. Lutsey et al. (17) yielded no statistically significant differences in the incidence of fracture between DOAC and DOAC among patients with AF. Further studies should confirm the association of DOAC with another DOAC in the risk of fracture.

The incidence of fracture position is an important factor that should be taken into consideration. Loss of bone quality due to aging and high incidence of osteoporotic fractures (especially hip and vertebral fractures) are the major threats to the elderly, causing significant morbidity, mortality, as well as high socioeconomic burdens (32, 33). AF itself is a risk factor for osteoporotic fractures. Overlapped risk factors such as older age, diabetes mellitus, and stroke are often shared by AF and osteoporotic fractures in patients, and they are also the risk factors for stroke (18). Thus, AF patients who take anticoagulants should be considered to be vulnerable to fractures. Our data suggested that DOACs usage is associated with a reduced incidence of overall fracture events. In addition, the benefits were also confirmed after patients were classified by the types of hip/pelvic fracture and the osteoporosis fracture rates, consistent with the results of the previous studies (24, 27).

### Limitations

We acknowledged that there are some limitations of this study. First, although we only included studies with the propensity score-matched or adjusted RRs, the quality of our meta-analysis was inherently limited because the potential unmeasured residual confounders would still exist due to the nature of real-world data. The high heterogeneity in this study might affect the reliability of findings, and further prospective studies should confirm our results. Second, only one study (23) provided the time within the therapeutic range value of warfarin users, which would underestimate the efficacy of warfarin. Third, the evaluation was limited to the AF patients treated with anticoagulants due to the limited data regarding patients with deep vein thrombosis or pulmonary vein thrombosis. Fourth, the age-related classifications of participants were also should be analyzed in this item's identification in further studies. Finally, due to the limited data of comparisons between DOAC vs. DOAC, we could not provide a choice of prescribing the most populated DOACs to AF patients especially those who are at a high risk of fractures.

## Conclusion

Our meta-analysis suggested that the use of DOACs was associated with a reduced risk of any fracture compared with VKAs. Further prospective studies should confirm these findings.

## Data Availability Statement

The original contributions presented in the study are included in the article/Supplementary Material, further inquiries can be directed to the corresponding authors.

## Author Contributions

All authors listed have made a substantial, direct and intellectual contribution to the work, and approved it for publication.

## Conflict of Interest

The authors declare that the research was conducted in the absence of any commercial or financial relationships that could be construed as a potential conflict of interest.

## References

[1] Luo G Ducy P McKee MD Pinero GJ Loyer E Behringer RR. Spontaneous calcification of arteries and cartilage in mice lacking matrix GLA protein. Nature. (1997) 386:78–81. 10.1038/386078a0 9052783

[2] Price PA Faus SA Williamson MK. Warfarin causes rapid calcification of the elastic lamellae in rat arteries and heart valves. Arterioscler Thromb Vasc Biol. (1998) 18:1400–7. 10.1161/01.ATV.18.9.1400 9743228

[3] Azuma K Shiba S Hasegawa T Ikeda K Urano T Horie-Inoue K. Osteoblast-specific gamma-glutamyl carboxylase-deficient mice display enhanced bone formation with aberrant mineralization. J Bone Miner Res. (2015) 30:1245–54. 10.1002/jbmr.2463 25600070

[4] Connolly SJ Ezekowitz MD Yusuf S Eikelboom J Oldgren J Parekh A. Dabigatran versus warfarin in patients with atrial fibrillation. N Engl J Med. (2009) 361:1139–51. 10.1056/NEJMoa0905561 19717844

[5] Granger CB Alexander JH McMurray JJ Lopes RD Hylek EM Hanna M. Apixaban versus warfarin in patients with atrial fibrillation. N Engl J Med. (2011) 365:981–92. 10.1056/NEJMoa1107039 21870978

[6] Patel MR Mahaffey KW Garg J Pan G Singer DE Hacke W. Rivaroxaban versus warfarin in nonvalvular atrial fibrillation. N Engl J Med. (2011) 365:883–91. 10.1056/NEJMoa1009638 21830957

[7] Giugliano RP Ruff CT Braunwald E Murphy SA Wiviott SD Halperin JL. Edoxaban versus warfarin in patients with atrial fibrillation. N Engl J Med. (2013) 369:2093–104. 10.1056/NEJMoa1310907 24251359

[8] Larsen TB Skjoth F Nielsen PB Kjaeldgaard JN Lip GY. Comparative effectiveness and safety of non-vitamin K antagonist oral anticoagulants and warfarin in patients with atrial fibrillation: propensity weighted nationwide cohort study. BMJ. (2016) 353:i3189. 10.1136/bmj.i3189 27312796 PMC4910696

[9] Nielsen PB Skjoth F Sogaard M Kjaeldgaard JN Lip GY Larsen TB. Effectiveness and safety of reduced dose non-vitamin K antagonist oral anticoagulants and warfarin in patients with atrial fibrillation: propensity weighted nationwide cohort study. BMJ. (2017) 356:j510. 10.1136/bmj.j510 28188243 PMC5421446

[10] Chao TF Liu CJ Lin YJ Chang SL Lo LW Hu YF. Oral anticoagulation in very elderly patients with atrial fibrillation: a nationwide cohort study. Circulation. (2018) 138:37–47. 10.1161/CIRCULATIONAHA.117.031658 29490992

[11] Liu X Xu Z Yu P Yuan P Zhu W. Non-vitamin K antagonist oral anticoagulants in secondary stroke prevention in atrial fibrillation patients: an updated analysis by adding observational studies. Cardiovasc Drug Ther. (2020) 34:569–78. 10.1007/s10557-020-06961-7 32297024

[12] Liao XZ Fu YH Ma JY Zhu WG Yuan P. Non-vitamin K antagonist oral anticoagulants versus warfarin in patients with atrial fibrillation and peripheral artery disease: a systematic review and meta-analysis. Cardiovasc Drugs Ther. (2020) 34:391–9. 10.1007/s10557-020-06962-6 32206988

[13] Gu ZC Zhou LY Shen L Zhang C Pu J Lin HW. Non-vitamin K antagonist oral anticoagulants vs. Warfarin at risk of fractures: a systematic review and meta-analysis of randomized controlled trials. Front Pharmacol. (2018) 9:348. 10.3389/fphar.2018.00348 29692734 PMC5903161

[14] Bengtson L Lutsey PL Chen LY MacLehose RF Alonso A. Comparative effectiveness of dabigatran and rivaroxaban versus warfarin for the treatment of non-valvular atrial fibrillation. J Cardiol. (2017) 69:868–76. 10.1016/j.jjcc.2016.08.010 27889397 PMC5411320

[15] Chan Y Lee H See L Tu H Chao T Yeh Y. Effectiveness and safety of four direct oral anticoagulants in Asian patients with nonvalvular atrial fibrillation. Chest. (2019) 156:529–43. 10.1016/j.chest.2019.04.108 31103697

[16] Binding C Bjerring OJ Abrahamsen B Staerk L Gislason G Nissen BA. Osteoporotic fractures in patients with atrial fibrillation treated with conventional versus direct anticoagulants. J Am Coll Cardiol. (2019) 74:2150–8. 10.1016/j.jacc.2019.08.1025 31648707

[17] Lutsey PL Norby FL Ensrud KE MacLehose RF Diem SJ Chen LY. Association of anticoagulant therapy with risk of fracture among patients with atrial fibrillation. JAMA Intern Med. (2019). 10.1001/jamainternmed.2019.5679 PMC6902159 31764956

[18] Lau WC Chan EW Cheung CL Sing CW Man KK Lip GY. Association between dabigatran vs warfarin and risk of osteoporotic fractures among patients with nonvalvular atrial fibrillation. JAMA. (2017) 317:1151–8. 10.1001/jama.2017.6912 28324091

[19] Higgins JP Altman DG Gotzsche PC Juni P Moher D Oxman AD. The cochrane collaboration's tool for assessing risk of bias in randomised trials. BMJ. (2011) 343:d5928. 10.1136/bmj.d5928 22008217 PMC3196245

[20] Moher D Liberati A Tetzlaff J Altman DG. Preferred reporting items for systematic reviews and meta-analyses: the PRISMA statement. PLoS Med. (2009) 6:e1000097. 10.1371/journal.pmed.1000097 19621072 PMC2707599

[21] Xue Z Zhang H. Non-vitamin K antagonist oral anticoagulants versus warfarin in asians with atrial fibrillation: meta-analysis of randomized trials and real-world studies. Stroke. (2019) 50:2819–28. 10.1161/STROKEAHA.119.026054 31422735 PMC6756252

[22] Zhu W Wan R Liu F Hu J Huang L Li J. Relation of body mass index with adverse outcomes among patients with atrial fibrillation: a meta-analysis and systematic review. J Am Heart Assoc. (2016) 5:e004006. 10.1161/JAHA.116.004006 27613773 PMC5079045

[23] Steffel J Giugliano RP Braunwald E Murphy SA Mercuri M Choi Y. Edoxaban versus warfarin in atrial fibrillation patients at risk of falling: Engage AF-TIMI 48 analysis. J Am Coll Cardiol. (2016) 68:1169–78. 10.1016/j.jacc.2016.06.034 27609678

[24] Norby FL Bengtson L Lutsey PL Chen LY MacLehose RF Chamberlain AM. Comparative effectiveness of rivaroxaban versus warfarin or dabigatran for the treatment of patients with non-valvular atrial fibrillation. BMC Cardiovasc Disord. (2017) 17:238. 10.1186/s12872-017-0672-5 28874129 PMC5585896

[25] Lucenteforte E Bettiol A Lombardi N Mugelli A Vannacci A. Risk of bone fractures among users of oral anticoagulants: an administrative database cohort study. Eur J Intern Med. (2017) 44:e30–1. 10.1016/j.ejim.2017.07.022 28734564

[26] Huang HK Liu PP Hsu JY Lin SM Peng CC Wang JH. Fracture risks among patients with atrial fibrillation receiving different oral anticoagulants: a real-world nationwide cohort study. Eur Heart J. (2020) 41:1100–8. 10.1093/eurheartj/ehz952 32006423

[27] Lau W Cheung CL Man K Chan EW Sing CW Lip G. Association between treatment with apixaban, dabigatran, rivaroxaban, or warfarin and risk for osteoporotic fractures among patients with atrial fibrillation: a population-based cohort study. Ann Intern Med. (2020) 173:1–9. 10.7326/M19-3671 32423351

[28] He N Dell'Aniello S Zhai S Suissa S Renoux C. Risk of fracture in patients with non-valvular atrial fibrillation initiating direct oral anticoagulants vs. vitamin K antagonists. Eur Heart J Cardiovasc Pharmacother. (2020). 10.1093/ehjcvp/pvaa094. [Epub ahead of print]. PMC8453296 32722764

[29] Fiordellisi W White K Schweizer M. A systematic review and meta-analysis of the association between vitamin K antagonist use and fracture. J Gen Intern Med. (2019) 34:304–11. 10.1007/s11606-018-4758-2 30511289 PMC6374254

[30] Fusaro M Dalle CL Dusso A Arcidiacono MV Valenti MT Aghi A. Differential effects of dabigatran and warfarin on bone volume and structure in rats with normal renal function. PLoS ONE. (2015) 10:e133847. 10.1371/journal.pone.0133847 26241483 PMC4524674

[31] Morishima Y Kamisato C Honda Y Furugohri T Shibano T. The effects of warfarin and edoxaban, an oral direct factor Xa inhibitor, on gammacarboxylated (Gla-osteocalcin) and undercarboxylated osteocalcin (uc-osteocalcin) in rats. Thromb Res. (2013) 131:59–63. 10.1016/j.thromres.2012.08.304 22999414

[32] Kim D Yang PS Kim TH Uhm JS Park J Pak HN. Effect of atrial fibrillation on the incidence and outcome of osteoporotic fracture- a nationwide population-based study. Circ J. (2018) 82:1999–2006. 10.1253/circj.CJ-17-1179 29794400

[33] Hippisley-Cox J Coupland C. Predicting risk of osteoporotic fracture in men and women in England and Wales: prospective derivation and validation of QFractureScores. BMJ. (2009) 339:b4229. 10.1136/bmj.b4229 19926696 PMC2779855

