# Supplementary material for: Association of Direct Oral Anticoagulants vs. Vitamin K Antagonists With Fractures in Atrial Fibrillation Patients: A Systematic Review and Meta-Analysis
Source: Front Cardiovasc Med. 2021 Jul 22;8:713187. doi: 10.3389/fcvm.2021.713187 (PMC8339256; doi:10.3389/fcvm.2021.713187)
Supplement: Supplementary file 1 [file Data_Sheet_1.doc]

**Supplementary Table 1. Search strategies determined until June 2021**

| **PubMed** | **Queries** | **Items** |
| --- | --- | --- |
| #1 | atrial fibrillation | 90,940 |
| #2 | atrial flutter | 9,822 |
| #3 | #1 OR #2 | 94,436 |
| #4 | non-vitamin K antagonist oral anticoagulants | 1,422 |
| #5 | direct oral anticoagulants | 7,042 |
| #6 | NOACs | 1,918 |
| #7 | DOACs | 2,187 |
| #8 | dabigatran | 5,892 |
| #9 | rivaroxaban | 6,628 |
| #10 | apixaban | 4,302 |
| #11 | edoxaban | 1,791 |
| #12 | #4 OR #5 OR #6 OR #7……OR #11 | 16,263 |
| #13 | vitamin K antagonists | 9,199 |
| #14 | warfarin | 31,591 |
| #15 | coumadin | 32,060 |
| #16 | phenprocoumon | 1,324 |
| #17 | acenocoumarol | 1,735 |
| #18 | #13 OR #14 OR #15 OR #16 OR #17 OR #18 | 40,648 |
| #19 | #3 AND #12 AND #18 | 4,984 |
| **EMBASE** |  |  |
| #1 | 'atrial fibrillation':ab,ti | 135,459 |
| #2 | 'atrial flutter':ab,ti | 9,549 |
| #3 | #1 OR #2 | 139,988 |
| #4 | 'non-vitamin K antagonist oral anticoagulants':ab,ti | 1301 |
| #5 | 'direct oral anticoagulants':ab,ti | 5628 |
| #6 | 'NOACs':ab,ti | 3810 |
| #7 | 'DOACs':ab,ti | 4107 |
| #8 | 'dabigatran':ab,ti | 10124 |
| #9 | 'rivaroxaban':ab,ti | 11742 |
| #10 | 'apixaban':ab,ti | 7829 |
| #11 | 'edoxaban':ab,ti | 2730 |
| #12 | #4 OR #5 OR #6 OR #7……OR #11 | 25303 |
| #13 | 'vitamin K antagonists':ab,ti | 7327 |
| #14 | 'warfarin':ab,ti | 41615 |
| #15 | 'coumadin':ab,ti | 2145 |
| #16 | 'phenprocoumon':ab,ti | 1391 |
| #17 | 'acenocoumarol':ab,ti | 1554 |
| #18 | #13 OR #14 OR #15 OR #16 OR #17 OR #18 | 50904 |
| #19 | #3 AND #12 AND #18 | 7002 |

**Supplemental Table 2. RRs and 95% CIs of fracture risks between DOACs versus VKAs in AF patients.**

|  | No. of reports | RRs and 95% CIs | *P*_interaction_ |
| --- | --- | --- | --- |
| **Any fracture** |  |  |  |
| DOACs | 10 | 0.80 [0.70, 0.91] | **-** |
| **DOAC types** |  |  | 0.16 |
| Dabigatran | 5 | 0.90 [0.80, 1.01] |
| Rivaroxaban | 4 | 0.73 [0.61, 0.88] |
| Apixaban | 3 | 0.75 [0.60, 0.92] |
| Edoxaban | 2 | 0.89 [0.77, 1.03] |
| **Fracture position** |  |  |  |
| Hip/pelvic fracture | 6 | 0.88 [0.79, 0.97] | 0.03 |
| Osteoporotic fracture | 3 | 0.63 [0.47, 0.84] |
| **Gender** |  |  |  |
| Males | 5 | 0.79 [0.67, 0.92] | 0.48 |
| Females | 5 | 0.71 [0.57, 0.89] |
| **Follow-up time** |  |  |  |
| ≥1 year | 6 | 0.76 [0.63, 0.91] | 0.84 |
| < 1 year | 2 | 0.73 [0.48, 1.10] |

AF=atrial fibrillation; DOACs=direct oral anticoagulants; VKAs=vitamin K antagonists; RR=risk ratio; CI=confidence interval.


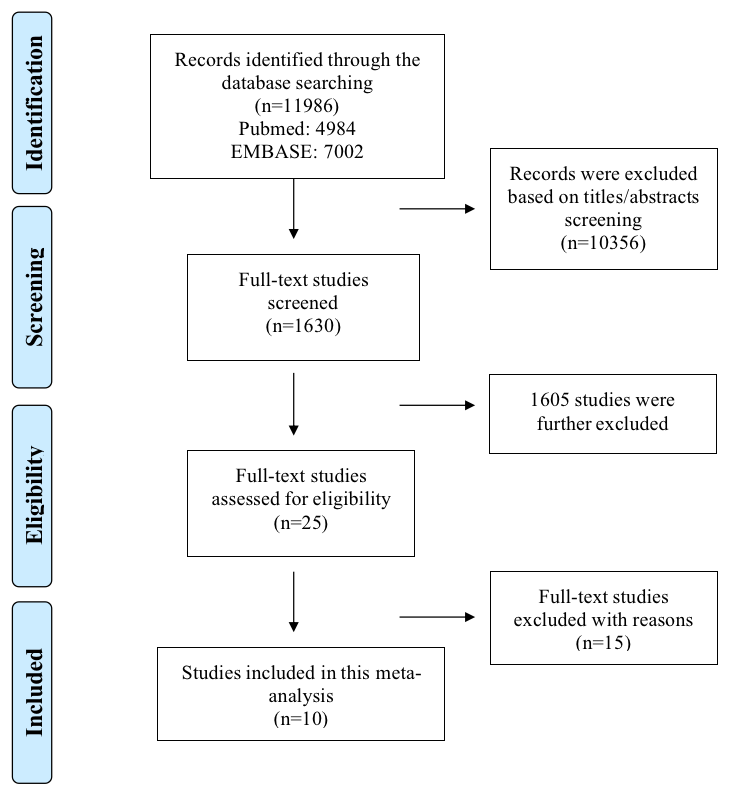


**Supplemental Figure 1. The process of electronic retrievals in this meta-analysis**


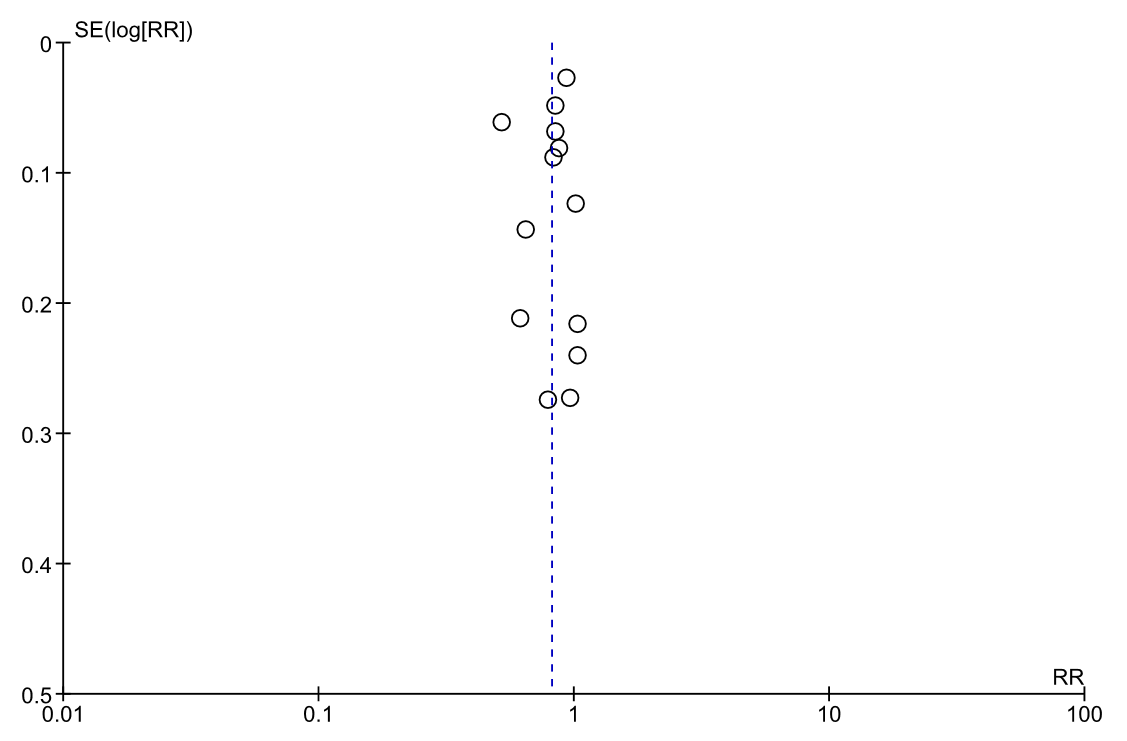


**Supplemental Figure 2. Funnel plots of all the included studies.**

Abbreviations: RR= risk ratio; SE=standard error.


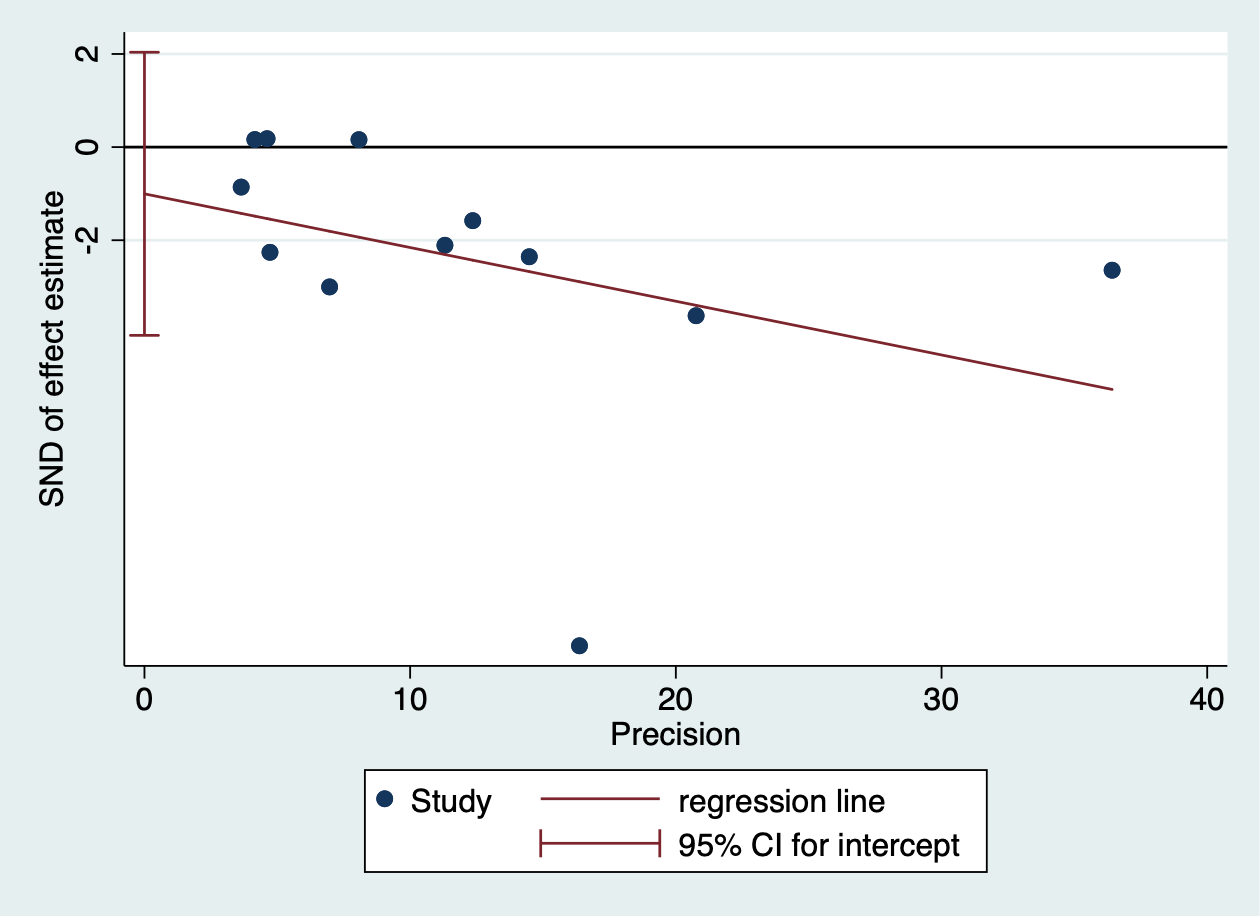


**Supplemental Figure 3. Egger’s plots of all the included studies.**
